# Two Modes of Cell Death Caused by Exposure to Nanosecond Pulsed Electric Field

**DOI:** 10.1371/journal.pone.0070278

**Published:** 2013-07-23

**Authors:** Olga N. Pakhomova, Betsy W. Gregory, Iurii Semenov, Andrei G. Pakhomov

**Affiliations:** Frank Reidy Research Center for Bioelectrics, Old Dominion University, Norfolk, Virginia, United States of America; National Research Council, Italy

## Abstract

High-amplitude electric pulses of nanosecond duration, also known as nanosecond pulsed electric field (nsPEF), are a novel modality with promising applications for cell stimulation and tissue ablation. However, key mechanisms responsible for the cytotoxicity of nsPEF have not been established. We show that the principal cause of cell death induced by 60- or 300-ns pulses in U937 cells is the loss of the plasma membrane integrity (“nanoelectroporation”), leading to water uptake, cell swelling, and eventual membrane rupture. Most of this early necrotic death occurs within 1–2 hr after nsPEF exposure. The uptake of water is driven by the presence of pore-impermeable solutes inside the cell, and can be counterbalanced by the presence of a pore-impermeable solute such as sucrose in the medium. Sucrose blocks swelling and prevents the early necrotic death; however the long-term cell survival (24 and 48 hr) does not significantly change. Cells protected with sucrose demonstrate higher incidence of the delayed death (6–24 hr post nsPEF). These cells are more often positive for the uptake of an early apoptotic marker dye YO-PRO-1 while remaining impermeable to propidium iodide. Instead of swelling, these cells often develop apoptotic fragmentation of the cytoplasm. Caspase 3/7 activity increases already in 1 hr after nsPEF and poly-ADP ribose polymerase (PARP) cleavage is detected in 2 hr. Staurosporin-treated positive control cells develop these apoptotic signs only in 3 and 4 hr, respectively. We conclude that nsPEF exposure triggers both necrotic and apoptotic pathways. The early necrotic death prevails under standard cell culture conditions, but cells rescued from the necrosis nonetheless die later on by apoptosis. The balance between the two modes of cell death can be controlled by enabling or blocking cell swelling.

## Introduction

Cell death induction by nsPEF has recently been proposed as a new therapeutic modality to ablate cancer. Cytotoxic efficiency of nsPEF against multiple cancer types has been demonstrated both *in vitro* [1]–[7] and *in vivo* [1], [5], [6], [8]–[10]. Interestingly, cancer cells reportedly were more vulnerable than matching normal cell lines [7]. Contemplated advantages of nsPEF over other ablation methods include higher probability of complete elimination of cancer cells; reduced collateral damage to healthy tissues and extracellular matrix; relative simplicity of the treatment; inhibition of angiogenesis; minimal systemic side effects; and fast recovery.

The exact mechanisms responsible for nsPEF cytotoxicity have been a subject of numerous studies [1]–[4], [11]–[17], but nonetheless remain elusive. Early studies in this area have noted fast and massive externalization of phosphatidylserine in nsPEF-treated cells, which was interpreted as a sign of apoptosis and a proof that apoptosis is the prevailing or even the sole mode of cell death after nsPEF [4], [18], [19]. As a result, the vast majority of studies into nsPEF-induced cell death focused solely on the apoptotic death pathway. Indeed, various types of cells exposed at lethal nsPEF doses expressed such manifestations of apoptosis as caspase activation, poly-ADP ribose polymerase (PARP) cleavage, cytochrome *C* release into the cytoplasm, and internucleosomal DNA fragmentation [4], [6], [12], [14]. The only type of necrosis considered in these studies was the so-called “secondary necrosis” (a final cell destruction following the apoptotic process *in vitro*).

However, the validity of PS externalization as a sign of apoptosis has been challenged with understanding that nsPEF opens pores in the cell plasma membrane. These pores could provide passage for calcium ions into the cell, causing scramblase activation and fast PS externalization [20], [21]. In addition, the pores can serve as a lipid-water interface pathway from the cell inside to the outside, allowing for calcium-independent lipid scrambling by a hypothetical “lateral drift” mechanism [22], [23]. In either case, the fast onset of PS externalization (<1 sec after nsPEF) suggested that this effect is not necessarily a step in the organized apoptotic process. These findings suggested that the conclusion about apoptosis prevalence after nsPEF (which was based primarily on the PS externalization data) may need to be revisited and revised.

Concurrently, several groups reported that nsPEF-treated cells typically swell [17], [24]–[27], which is a morphological hallmark of necrosis. Permeabilization of the cell plasma membrane was identified as the principal cause of the necrotic cell transformation [17], [24]. Recently we reported that a significant fraction of nsPEF-treated Jurkat and U937 cells died at intervals much shorter than what it typically takes to complete the apoptotic process [12]. The number of “live” cells (impermeable to Trypan blue) decreased almost twofold already at 2 hr after the nsPEF exposure (600 pulses, 10 ns, 100 kV/cm), but the internucleosomal DNA fragmentation developed only in 3 hr; hence, a large fraction of cells died before reaching this apoptotic step. Significant cell death could be observed in the absence of PARP cleavage, suggesting a caspase-independent mechanism [28]. In agreement with the above findings, Yin et al. [6] observed destroyed cells and cell fragments after intense nsPEF treatments *in vitro*, and interpreted it as a necrotic effect of exposure. Several studies have reported both apoptotic and necrotic cell death after nsPEF treatments of tumors *in vivo* [9], [29].

In this study, we show that cell swelling and membrane rupture are the predominant mechanisms of the early cell death following nsPEF exposure. The prevalence of the early necrotic death was characteristic for nsPEF treatments with either “long” 300-ns pulses or “short” 60-ns pulses, within a wide range of doses, and for diverse pulse delivery protocols. This primary necrotic death prevented the development and observation of apoptosis in nsPEF-treated cells. However, the inhibition of the primary necrosis led to a much higher incidence of delayed cell death by apoptosis.

## Materials and Methods

### Cells and Media

Experiments were performed in a suspension cell line U937 (human monocytes). The cells were obtained from ATCC (Manassas, VA) and propagated at 37°C with 5% CO_2_ in air in RPMI-1640 medium supplemented with 10% fetal bovine serum, 2 mM L-glutamine, 100 IU/ml penicillin, and 0.1 µg/ml streptomycin. The media and its components were purchased from Mediatech Cellgro (Herdon, VA) except for serum (Atlanta Biologicals, Norcross, GA). Other chemicals used for this study were from Sigma–Aldrich (St. Louis, MO) unless noted otherwise.

### Modifications of the Growth Medium to Inhibit nsPEF-induced Cell Swelling

The nsPEF-induced water uptake is driven by the colloid-osmotic mechanism and can be blocked by the presence of a nanopore-impermeable solute such as sucrose [26]. Importantly, this effect is achieved without changing the integral osmolality of the extracellular medium.

In this study, the RPMI medium containing sucrose (hereinafter, “RPMI+sucrose”) was produced by mixing RPMI (containing cells and all supplements listed above) with an isoosmotic (290 mOsm/kg) water solution of sucrose. Mixing was performed at the proportion of 4∶1 or 7∶3, yielding fractional osmolalities due to the sucrose of 58 or 87 mOsm/kg, respectively. As found in preliminary experiments (data not shown), such fractions of sucrose provided an accurate colloid-osmotic balance to the cytosol, thereby preventing any volume changes in cells permeabilized by nsPEF.

An unavoidable side effect of the isoosmotic mixing of RPMI with sucrose was the dilution of nutrients, salts, serum, and other ingredients of the medium. This dilution was well tolerated by cells, although could cause a minor slowdown of the propagation rate. Nonetheless, in order to match this dilution, the parallel control samples were diluted by an isoosmotic NaCl solution at the same proportions (“RPMI+NaCl”). Na^+^ and Cl^-^ ions are small solutes capable of passing the nanopores and therefore do not prevent the water uptake [25], [26]. As shown below, the growth rate of control U937 cells (not exposed to nsPEF) in RPMI+sucrose was the same as in RPMI+NaCl.

In several sets of experiments, the parallel control samples were diluted with fresh RPMI instead of NaCl (“RPMI+RPMI”). In such cases, cells were left in RPMI+sucrose and in RPMI+RPMI only for a brief time interval (e.g., 30 min). Afterwards, all samples were diluted 10x with fresh RPMI, thereby canceling out any differences in the medium composition.

The exact protocols that were employed for each specific experiment are described in the Results section and in figure captions. As shown below, the RPMI+sucrose medium always modified the effects of nsPEF in a similar way, irrespective of the specific protocol employed.

### nsPEF Exposure Methods and Protocols

In most experiments, we used trapezoidal pulses of 300 ns duration from an AVTECH AVOZ-D2-B-ODA generator (AVTECH Electrosystems, Ottawa, Ontario, Canada). Pulse trains of needed duration at a selected repetition rate of 200 Hz were triggered externally from a model S8800 stimulator (Grass Instruments Co., Quincy, MA). Pulses were delivered to an electroporation cuvette with cells using a 50- to 10-Ohm transition module (AVOZ-D2-T, AVTECH Electrosystems) modified into a cuvette holder. The pulse amplitude and shape were monitored using a Tektronix TDS 3052B oscilloscope. More details of this exposure procedure were reported earlier [30].

Main findings of this study were replicated using 60-ns pulses from a high-voltage home-made pulse generator described previously [4], [31]. The goal for testing 60-ns pulses and different exposure protocols was to demonstrate that the established mechanisms of cell death hold true for diverse nsPEF treatments rather than just for a specific, randomly chosen type of treatment. The 60-ns pulse generator utilizes the pulse forming network technology and a simple spark gap in the atmospheric air works as a switch. With this device, the pulse repetition rate can only be approximately controlled by the rate of network charging. We utilized the pulse rate of about 1 Hz, and the number of pulses delivered to the sample was controlled manually. Because of multiple differences in the pulse delivery protocols for 300- and 60-ns pulses, any quantitative comparison between these treatments was not intended.

For nsPEF exposure, cells were harvested during the logarithmic growth phase, pelleted by centrifugation, and resuspended in a fresh growth medium. As found in preliminary experiments, the cell density at the time of exposure within the range from 0.5 to 8×10^6^ cells/ml did not affect the nsPEF efficiency (data not shown). For cell survival studies, the cell density at the time of exposure was 0.6 or 1.2×10^6^ cells/ml; for Western blot measurements which required larger cell quantities, the density was increased to 7×10^7^ cells/ml.

Immediately following nsPEF exposure, cells were diluted to 0.2–0.7×10^6^ cells/ml into RPMI+RPMI, RPMI+sucrose, or RPMI+NaCl medium and stored in the incubator until further measurements or manipulations.

In several early series of experiments, cells were placed in the modified medium prior to nsPEF exposure. Although slightly lower electrical conductance of RPMI+sucrose compared to other media could affect the efficiency of nsPEF, we observed no differences compared to post-exposure dilutions.

In any series of experiments, samples in different media and/or exposed to different nsPEF parameters were handled in exactly the same manner, and different treatments were applied in a random sequence. All series were also accompanied by parallel sham-exposed controls.

NsPEF exposures were performed at a room temperature of 22–24°C. Heating of cell samples by nsPEF did not exceed 7°C, as measured with a fiber optic ReFlex-4 thermometer (Nortech Fibronic, Quebec City, Canada).

### Viability Assays

Cell survival was measured at different times after nsPEF exposure using either MTT (3-(4,5-Dimethylthiazol-2-yl)-2,5-diphenyltetrazolium bromide) assay or a fluorescent dye exclusion/quenching method (AO/PI assay). Both assays were described in detail previously [30].

In brief, for the MTT assay (BioAssay Systems, Hayward, CA) we used a 96-well format and a Synergy 2 microplate reader (BioTEK, Winooski, VT). 10 µl of the MTT reagent were added to 100 µl of cell suspension and incubated for 4 hr until adding the solubilization buffer. The plates were left on an orbital shaker overnight and the absorbance was read at 570 nm.

The AO/PI assay utilized a mixture of a membrane-permeable dye acridine orange (AO) and a membrane-impermeable dye propidium iodide (PI). This method detected only massive PI uptake characteristic for dead cells with fully ruptured plasma membrane. Immediately prior to measurements, a 20 µl aliquot of the cell suspension was mixed with the equal volume of staining solution (0.5 µg/ml AO and 100 µg/ml PI in a phosphate-buffered saline, PBS). The sample was loaded into a counting chamber of the automated cell counter Cellometer Vision with two-channel cell fluorescence detection (Nexcelom Bioscience LLC, Lawrence, MA). Live cells were distinguished by bright AO fluorescence (exc./em. 475/535 nm). In cells with compromised membrane AO emission was quenched by PI uptake. Combined fluorescence of either AO or PI (exc./em. 525/595 nm) was used to determine the total (live+dead) cell counts.

### Cell Diameter Measurement

Cells in the counting chamber of the Cellometer were imaged in bright field, automatically de-clustered and distinguished from debris. The automated recognition of cells in the sample was verified manually and corrected if needed. The diameters of 400–600 cells per sample were automatically measured from the image and logged using Cellometer software.

### Microscopy

Cell images were taken using an Olympus IX71 inverted microscope equipped with a Retiga 2000R Fast 1394 CCD camera (QImaging, Surrey, BC, Canada). We used 5 µg/ml PI and 1 µM YO-PRO-1 dye (Life Technologies, Grand Island, NY) as fluorescent markers of membrane permeabilization. The dyes were added about 10 min prior to scheduled measurements, and cells were allowed to settle down. Images taken with a 20x, 0.40 NA dry objective were captured and processed with MetaMorph 7.5.2 software (Molecular Devices, Foster City, CA).

For a more detailed but mostly qualitative analysis of morphological effects of nsPEF, we used an Olympus FluoView 1000 confocal scanning system. It utilized an IX81 microscope equipped with differential-interference contrast (DIC) optics. 10-µl aliquots of cells with the dyes already added were placed on a coverslip, allowed to settle down for 5–10 min, and then examined with a 40x, 0.95 NA objective. Due to the small size of the sample and varied times for cell settling, the images were not used for cell scoring.

### Caspase 3/7 Activity

We utilized a Caspase- Glo®3/7 Assay from Promega Corporation (Madison, WI) according to manufacturer’s instructions. Briefly, cells were exposed at 7×10^6^ cells/ml and diluted tenfold into RPMI+sucrose or RPMI+NaCl. The cells were incubated at 37°C in 5% CO_2_ humidified air. In 1, 2, 6, and 24 hr after nsPEF, cells were aliquoted in triplicate at 50 µl/well into a 96-well plate; 10 µl of Caspase- Glo®3/7 reagent were added to each well, and the plate was briefly mixed on an orbital shaker. After 40 min of incubation at room temperature, the level of luminescence was measured by the Synergy 2 reader.

U937 cells incubated with 10 µM staurosporin were used as a positive control for the induction of apoptosis.

### Immunoblot Analysis and Quantitation of Poly-ADP Ribose Polymerase (PARP) Cleavage

Specific PARP cleavage is an established hallmark of apoptosis [32], [33]. Both the full-length 116 kDa PARP and its 89 kDa fragment can be detected together by immunoblotting. Quantitation of the apoptotic fraction of cells from the relative amounts of intact and cleaved PARP is intrinsically ratiometric and therefore more quantitative than most of comparable assays.

At 1, 2, 6, or 9 hrs after nsPEF exposure, samples containing approximately 4×10^5^ cells were chilled on ice and pelleted by centrifugation. The pellet was washed twice with ice-cold PBS and lysed in 30 µl of a buffer containing 20 mM HEPES (pH 7.5), 200 mM NaCl, 10 mM EDTA, 1% Triton X-100, and freshly added 1 mM DTT (dithiothreitol), 10 µM Leupeptin, 1 mM PMSF (phenylmethanesulfonyl fluoride), and 0.2 mg/ml Benzamidin. The samples were vortexed and centrifuged at 15,000 g at 4°C for 10 min. The supernatant containing PARP was stored at −80°C.

Proteins were separated by electrophoresis on a NuPAGE 4–12% Bis-Tris SDS-polyacrylamide gel (Life Technologies) and then transferred to Immun-Blot Low Fluorescence PVDF membrane (Bio-Rad Laboratories, Hercules, CA) by wet electroblotting at 30 volts for 1 hr. Odyssey marker IRDye 680/800 was added as a molecular weight standard (LI-COR Biosciences, Lincoln, NE). The blots were blocked by incubation overnight at 4°C in the Odyssey blocking buffer (LI-COR Biosciences).

Rabbit anti-PARP primary polyclonal antibodies (Roche Diagnostics GmbH, Mannheim, Germany) were diluted 1∶2,000 in the Odyssey blocker with 0.2% Tween-20. Donkey anti-rabbit IgG(H+L) secondary antibodies conjugated with an infra-red fluorophore IRDye-680LT (LI-COR Biosciences) were diluted 1∶20,000 in the same buffer. The blots were treated with the primary antibodies for 1 hr at room temperature, washed 4 times (5 min each) in PBS with 0.1% Tween-20, treated with secondary antibodies for 1 hr, and washed again.

The membranes were imaged using Odyssey 9120 Infrared Imaging System (LI-COR Biosciences) in the 700 nm channel. The images were quantified using MetaMorph software (Molecular Devices).

The fraction of the cleaved PARP (*K,* %) was measured as: 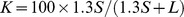 where *L* and *S* are the fluorescence intensities of the 116 kDa full-length PARP and of the 89 kDa PARP fragment, respectively. The coefficient 1.3 was used for *S* mass correction. The quantitative data from 4–5 independent experiments were processed for each timepoint and for each type of nsPEF treatment. Staurosporin-induced apoptosis was used as a positive control.

### General Protocols and Statistics

All of experiments were designed to minimize potential biases and to ensure the accuracy and reproducibility of results. All experiments included a sham-exposed parallel control group, which was subjected to all the same manipulations and procedures as the nsPEF-exposed samples, excluding only the nsEP exposure itself. Various regimens of the nsPEF treatment and parallel control experiments alternated in a random manner, and no “historical” controls were accepted. Diverse buffer conditions were also tested in parallel. When measurements were made in triplicate (e.g., cell viability using MTT assay), the mean of the three values was counted as a single experiment. To achieve statistical significance, we usually ran 4–6 independent experiments per each group (a minimum of 3). Student’s *t*-test with Dunnet’s correction when applicable [34], [35] was employed to analyze the significance of differences. The data were presented in the graphs as mean values +/− s.e. The difference at *p*<0.05 level (2-tailed) was regarded as statistically significant. Due to multiple statistical comparisons made (exposures versus controls; different buffers; different timepoints; etc) we chose to let the error bars speak for the statistical difference with minimum use of special symbols. For clarity, the special symbols were only used for the RPMI+sucrose groups, to indicate the significant difference from the RPMI+NaCl group (*) and from the sham-exposed control (#). In fact, most effects reported below were quite robust and statistically significant at *p*<0.01 or better at least for several timepoints.

## Results

### Sucrose Inhibits nsPEF-induced Cell Swelling and Prevents Membrane Rupture

In a recent study [26], we showed that 60- as well as 600-ns pulses cause water uptake and cell swelling due to the colloid-osmotic imbalance [25], [36]–[38]. In brief, the water uptake results from the fact that small solutes can diffuse through membrane pores towards the concentration equilibrium, whereas the larger solutes cannot. Hence, the larger solutes remain trapped inside the cell, thereby creating an osmotic gradient to attract water. This gradient can be counterbalanced by replacing small solutes (such as Na^+^ and Cl^-^) in the bath buffer with larger, pore-impermeable solutes such as sucrose. Such replacement prevents cell swelling even though the osmolality of the extracellular buffer remains unchanged [26].

**Figure 1:**
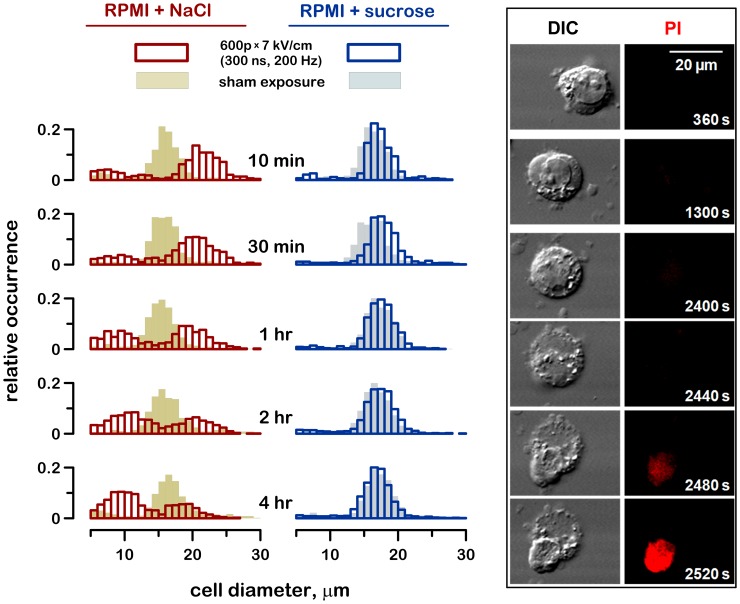
Sucrose inhibits cell swelling and membrane rupture caused by nsPEF. The bar charts show the frequency distribution of cell diameter values at the indicated time intervals after nsPEF exposure and in sham-exposed controls. Cells were exposed in the RPMI medium and placed immediately afterwards into either RPMI+NaCl or RPMI+sucrose (87 mOsm/kg due to sucrose); see text for more details. 400–600 cells per group were measured at each timepoint. Note fast cell swelling followed by membrane rupture and apparent shrinkage in RPMI+NaCl, but not in the RPMI+sucrose. Representative cell images in the differential interference contrast (DIC) and propidium iodide (PI) fluorescence channels illustrate swelling and eventual membrane rupture in the RPMI+NaCl medium.

Fig. 1 shows a typical time dynamics of cell volume changes following nsPEF treatment. All cell samples were exposed to 600 pulses (300-ns pulse duration, 7 kV/cm, 200 Hz) in a standard RPMI medium. Immediately after the exposure, the samples were mixed 7∶3 with isoosmotic NaCl or sucrose as described above. Sham-exposed cell samples that served as controls were diluted the same way.

The diameter of control cells did not depend on the time of incubation after nsPEF or on whether the sucrose or NaCl was added to the medium. The distribution of cell diameters was bell-shaped, with the peak at 16–18 µm. NsPEF exposure caused rapid swelling in the RPMI+NaCl group, eventually followed by membrane rupture and cell destruction. The destroyed cells shrunk abruptly, almost to the size of the nucleus, so the cell death was manifested as an increased fraction of smaller cells. This mode of cell death was essentially identical to the classic scenario of hemolysis caused by the electroporation of erythrocytes [38].

Consistent with the earlier observations [26], the dilution of RPMI with sucrose fully prevented cell swelling (Fig. 1, right column). Consequently, sucrose also prevented the secondary cell shrinkage due to the membrane rupture.

### Dual Effect of Sucrose on Cell Survival

**Figure 2:**
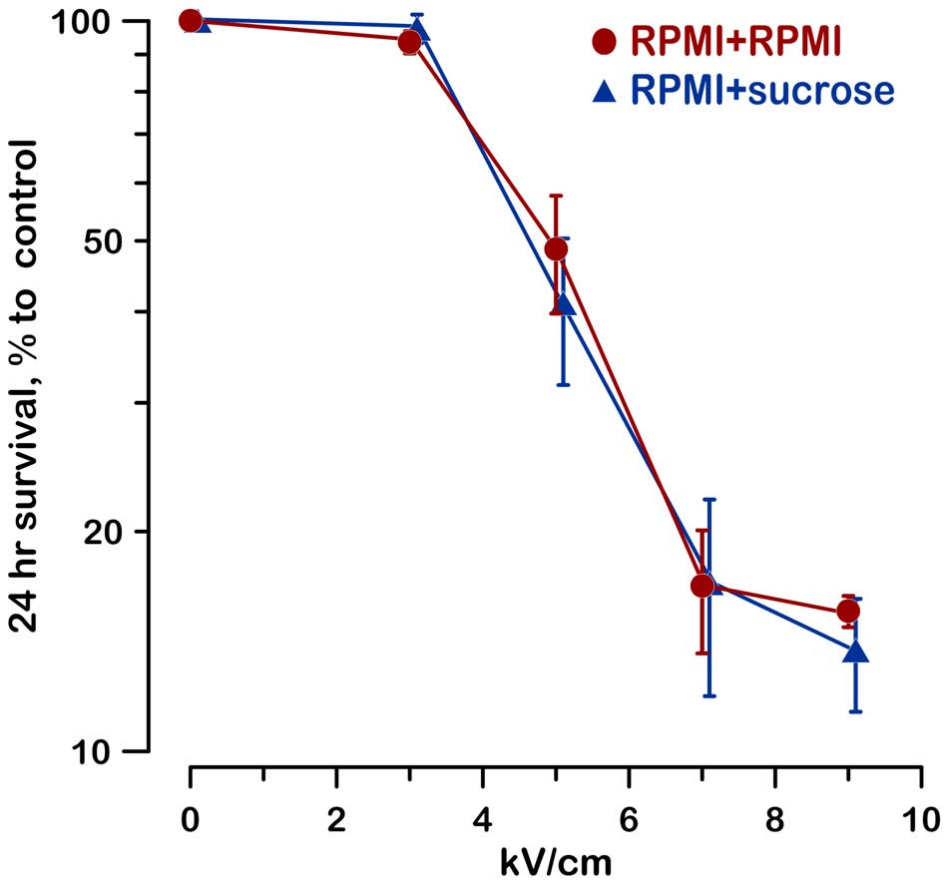
Lack of the effect of sucrose on the 24-hr survival of nsPEF-treated cells. Cells in RPMI were mixed with sucrose (RPMI+sucrose; 60 mOsm/kg due to sucrose) or fresh RPMI (RPMI+RPMI) before nsPEF exposure (600 pulses, 300-ns). At 30 min after the exposure, all samples were diluted tenfold with fresh RPMI and incubated until measuring cell survival by the MTT assay at 24 hr (mean values +/− s.e. for 6 independent experiments).

While it was most logical to expect that the inhibition of cell swelling and prevention of membrane rupture by sucrose should improve cell survival, the experiments showed that it was not the case. At 24 hr after nsPEF exposure, and for a wide range of exposure intensities, the cell survival stayed remarkably the same in the presence or absence of sucrose (Fig. 2).

**Figure 3:**
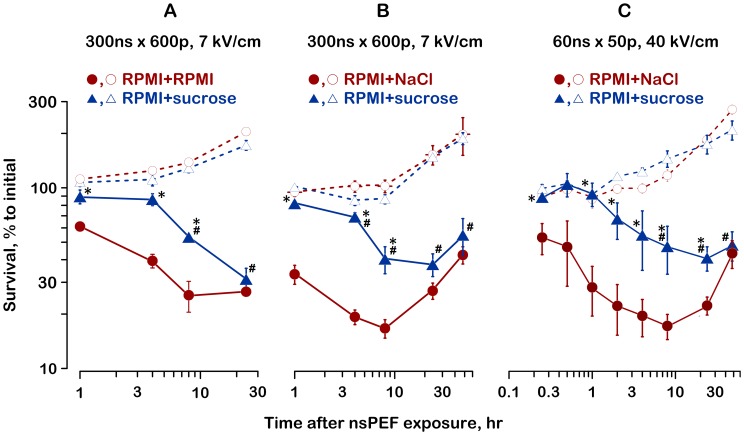
Inhibition of swelling improves the short-term but not the long-term survival after nsPEF exposure. Panels A, B, and C represent the data from three independent sets of experiments performed under different exposure conditions and using different protocols. For panel A, cells in RPMI were mixed with sucrose (RPMI+sucrose; 60 mOsm/kg due to sucrose) or fresh RPMI (RPMI+RPMI) before nsPEF exposure (the same protocol as in Fig. 2). For panels B and C, cells were exposed in the RPMI and placed immediately afterwards into either RPMI+NaCl or RPMI+sucrose (87 mOsm/kg due to sucrose), same as in Fig. 2. See graph legends and text for more details. Cell survival was measured by the AO/PI assay and normalized to the pre-exposure value (mean+/− s.e., n = 4–6). Cell survival in sham-exposed controls is shown by dashed lines and open symbols. * *p*<0.05 for the difference of RPMI+sucrose from RPMI+NaCl (or RPMI+RPMI); # *p*<0.05 for the difference of RPMI+sucrose from the respective sham-exposed control. Other significant differences are not shown for clarity.

This unexpected finding has stimulated the analyses of the time course of nsPEF-induced cell death under diverse conditions (Fig. 3). Panels A, B, and C represent three independent series of experiments. In all these series, cell survival was monitored by the AO/PI assay for up to 24 hr (A) or 48 hr (B and C) after the nsPEF exposure. For experiments in panel A, cells in RPMI were diluted with either sucrose or fresh RPMI prior to nsPEF treatment. For experiments in panels B and C, the dilution with either sucrose or NaCl was performed immediately after the exposure. The nsPEF exposure was either 600 pulses, 300 ns, 200 Hz at 7 kV/cm (panels A and B), or 50 pulses, 60 ns, approximately 1 Hz at 40 kV/cm (panel C).

Irrespective of the methodological differences, the effects observed in these experiments were similar. In sham-exposed cells, modifications of the growth medium had little or no effect: cells grew similarly in RPMI+sucrose and in RPMI+NaCl (panels B and C), and perhaps slightly faster in the RPMI (panel A). At the same time, the presence of sucrose profoundly improved the survival of nsPEF-exposed cells at early time intervals (1–8 hr). However, at the later time intervals the cells protected by sucrose continued to die, whereas those without sucrose protection already started to recover. Eventually, the percent of viable cells became the same, and the protective effect of sucrose was nullified.

**Figure 4:**
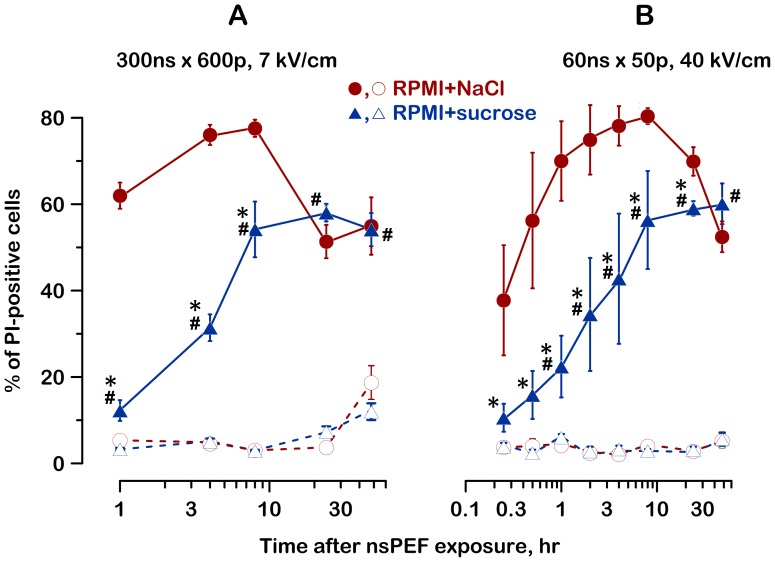
Inhibition of swelling blocks the early cell death after nsPEF. Dead cells were identified by the AO/PI assay. The total number of cells counted at each timepoint was taken as 100% (mean+/− s.e., n = 4–6). The data in panels A and B are from the same experiments as in Fig. 3, B and C. See text and Fig. 3 for details. PI uptake in sham-exposed controls is shown by dashed lines and open symbols.

This phenomenon can also be illustrated by measuring the fraction of dead (PI-positive) cells at different times after nsPEF exposure (Fig. 4). Without the sucrose protection, the fraction of dead cells increased rapidly to its maximum at 8–10 hr, and gradually decreased afterwards. With the sucrose protection, the fraction of PI-positive cells increased after a delay of several hours, but showed no reduction within the period of observation.

To summarize, the presence of sucrose efficiently inhibited the early cell death (just as expected from the blockage of cell swelling), but the rescued cells nonetheless died later on because of some other reason. As a result, the cell survival at intervals of 24 and 48 hr was not significantly improved by the inhibition of cell swelling (Figs. 2 and 3).

### Blockage of Cell Swelling Switches the Cell Death Pathway from Necrosis to Apoptosis

**Figure 5:**
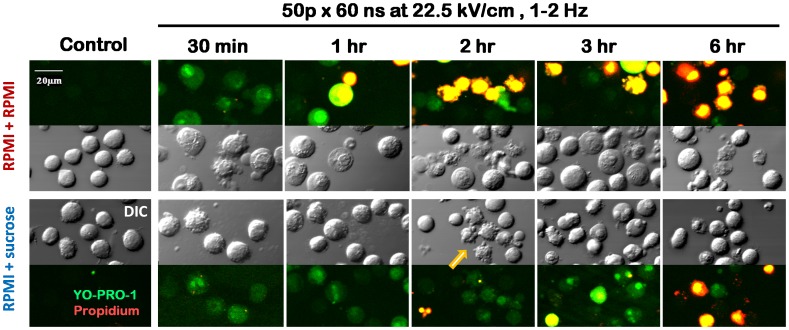
Effects of sucrose on cell swelling and membrane permeability. DIC and fluorescence images of nsPEF-exposed cells incubated in either RPMI+RPMI or RPMI+sucrose. Green: YO-PRO-1; red: PI; yellow: both dyes overlapped. Parameters of exposure and times after it are given in the legend. Cells were handled the same way as in Fig. 2 but without additional dilution at 30 min. The dyes were added 5–10 min prior to taking an image. Note early cell swelling and rupture in the RPMI+RPMI but not in the RPMI+sucrose medium. The survivors show no YO-PRO-1 uptake in the RPMI+RPMI, but remain permeable to the dye in the RPMI+sucrose group. An arrow points to a group of cells that display the apoptotic blebbing and fragmentation.

The cell survival data reported above were consistent with gradual changes in the cell appearance and the uptake of membrane-impermeable dyes with time after nsPEF (Fig. 5).

At 0.5–1 hr after the exposure, cells left in the RPMI were swollen and developed necrotic-type blebs (also sometimes called “blisters”). In the presence of sucrose, nsPEF-exposed cells displayed few if any morphological changes. In both these cell populations, the plasma membrane was partially compromised, allowing the uptake of YO-PRO-1 but not of PI (propidium ion is larger than YO-PRO-1 and has low permeability through nsPEF-opened pores [25], [26], [39]). Later on, membrane rupture in swollen cells resulted in massive PI uptake and dual staining of dead cells by both Yo-PRO-1 and PI. However, cells that did not rupture became impermeable to YO-PRO-1 and regained the normal appearance. At 4 and 6 hr after nsPEF, most of cells in RPMI were either dead (double-stained) or normal (no staining). In the presence of sucrose, many cells developed cytoplasm fractionation (apoptotic blebbing) and remained permeable to YO-PRO-1. These manifestations suggested that the delayed death in sucrose-rescued cells could be a result of apoptosis.

**Figure 6:**
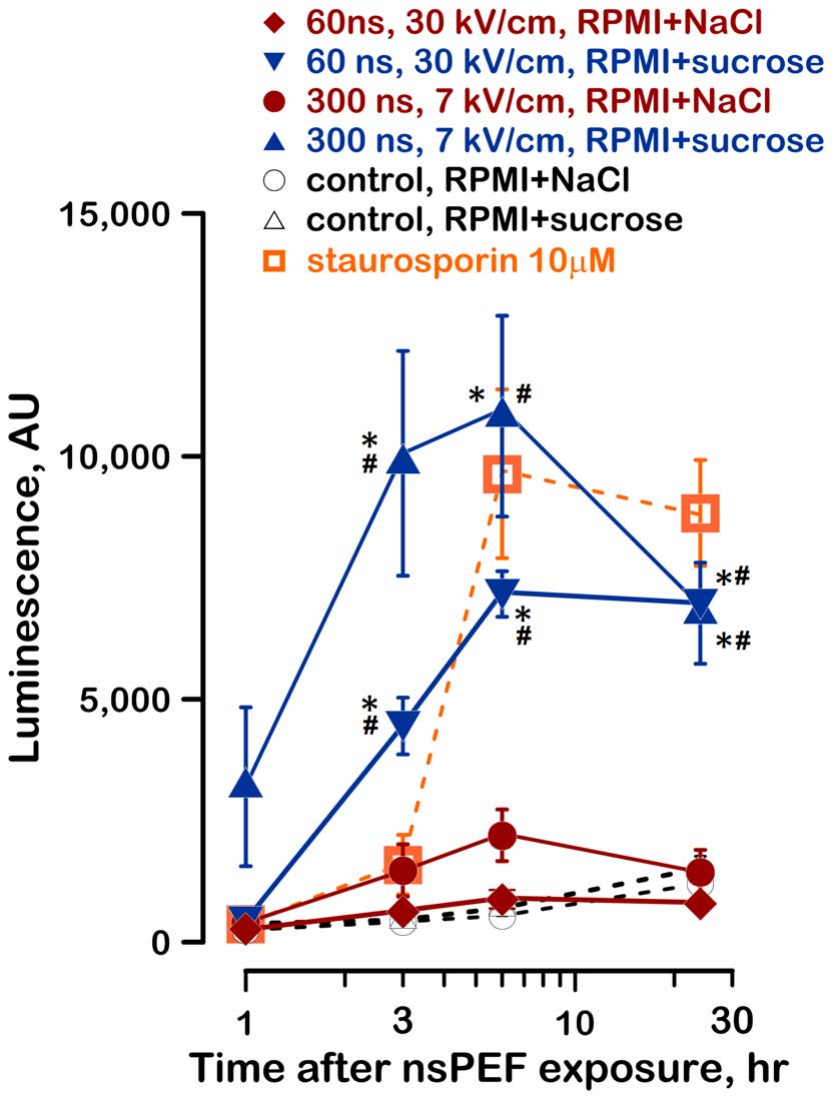
Inhibition of swelling in nsPEF-exposed cells facilitates caspase 3/7 activation. The exposure parameters and media are identified in the legend. Growth media were changed the same way as in Fig. 1. Caspase-3/7 was measured by a luminescence assay. For a positive control, apoptosis was induced by 10 µM of staurosporin. Mean values +/− s.e. for n = 3. See text and Fig. 3 for details.

**Figure 7:**
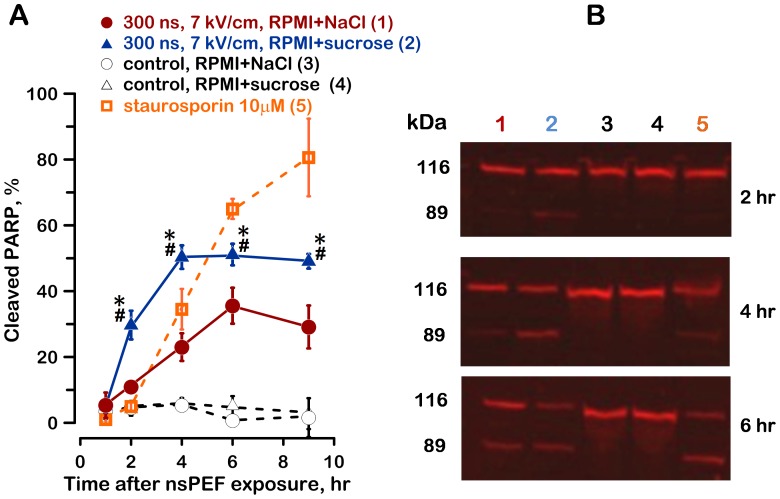
PARP cleavage in nsPEF-exposed cells. A: The fraction of cleaved PARP is increased when nsPEF-exposed cells are protected with sucrose. Mean values +/− s.e. for n = 4–5. Growth media were changed the same way as in Fig. 1. NsPEF and media conditions are specified in the legend. The numbers in parentheses correspond to the lanes in panel B, which shows representative Western blots for intact and cleaved PARP (116 and 89 kDa, respectively). See text and Fig. 3 for details.

Indeed, protection of nsPEF-treated cells by sucrose caused profound activation of caspase 3/7 already at 1 hr after the exposure, reaching maximum at 3–6 hr (Fig. 6). The cells left in RPMI+NaCl after nsPEF exposure showed just minor caspase activation. These findings were corroborated by another hallmark of apoptosis, namely the markedly increased cleavage of PARP (Fig. 7). The data looked similar for exposures to 300- and 60-ns pulses; taken together with the cell swelling and survival measurements, the data indicated that the cell damage and cell death mechanisms caused by 300- and 60-ns pulses were similar.

Figs. 6 and 7 also include the data for a chemically-induced apoptosis as a positive control. We used staurosporin, a well-established agent which, at the tested concentration, caused apoptotic cell death in almost 100% of U937 cells (data not shown). Interestingly, caspase 3/7 activation and PARP cleavage in the staurosporin-induced apoptosis developed as much as 2–3 hr later as compared to the nsPEF-induced apoptosis. One possible interpretation of this observation is that nsPEF just “bypassed” the initial steps of the staurosporin-induced apoptotic cascade [40]. At the same time, we showed earlier that the internucleosomal DNA fragmentation in nsPEF-exposed cells developed later than in heat-shocked cells [12]. Overall, the time course of the nsPEF-induced apoptosis appeared within the time limits reported for other apoptotic factors.

### The Balance between the Two Modes of Cell Death

**Figure 8:**
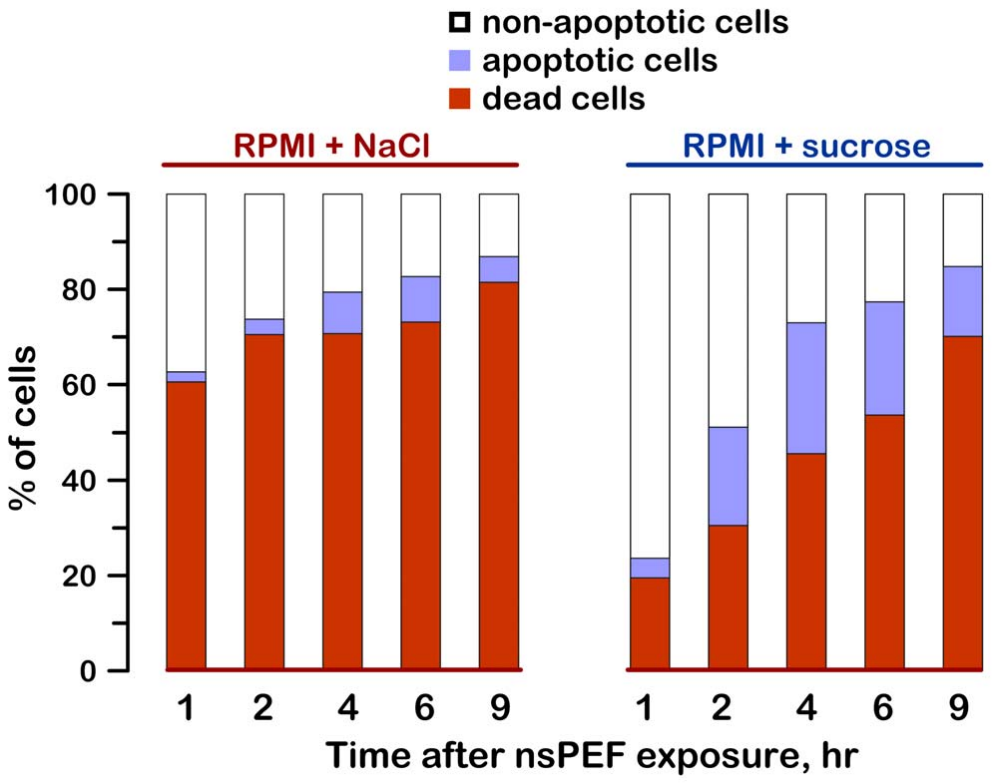
The structure of nsPEF-exposed cell populations with and without blockage of cell swelling with sucrose. Bars show relative fractions of non-apoptotic, apoptotic, and dead cells at different timepoints after nsPEF (600 pulses, 300 ns, 7 kV/cm). Growth media were changed the same way as in Fig. 1. Dead and live cells were counted by the AO/PI assay. The fraction of apoptotic cells among live cells was considered proportional to the fraction of cleaved PARP. The data were averaged from 4–5 experiments; the error bars are omitted for clarity.

**Figure 9:**
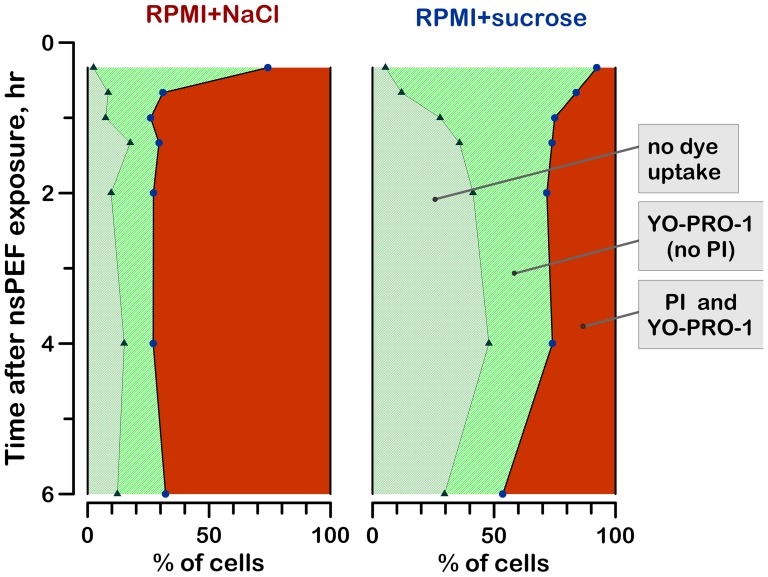
The effect of sucrose on Yo-PRO-1 and PI uptake by nsPEF-exposed cells. Growth media were changed after nsPEF exposure (600 pulses, 300 ns, 7 kV/cm) the same way as in Fig. 1. The number of cells displaying no dye uptake, YO-PRO-1 uptake, and both YO-PRO-1 and PI uptake were automatically counted in microscope images as described in Methods. The total number of cells counted in each sample was taken as 100%. The PI-positive cells were presumed dead. YO-PRO-1-positive cells could be either apoptotic or just transiently permeabilized to this dye by nsPEF. Cells negative for either dye were regarded as live, non-apoptotic. The data were averaged from 3 experiments; error bars are omitted for clarity.

The proportion of necrotic, apoptotic, and non-apoptotic live cells, as determined by different approaches, is presented in Figs. 8 and 9.

In Fig. 8, the fraction of PI-positive (dead) cells was determined by the AO/PI assay. The remaining fraction of live cells was split into the “apoptotic” and “non-apoptotic” subpopulations based on the relative amounts of intact and cleaved PARP measured in the same sample. With time after nsPEF exposure, non-apoptotic cells could shift into either “apoptotic” or “dead” categories, and the “apoptotic” cells could also become “dead”.

After exposure to 600 pulses (300 ns, 7 kV/cm), 60% of cells were already dead at 1 hr if kept in the RPMI+NaCl medium. Taking into account the early occurrence of the cell death, morphological signs (cell swelling and membrane rupture), and the lack of concurrent caspase 3/7 activation or PARP cleavage, the early cell death can be categorized as a primary necrosis. Later on, a fraction of cells kept in RPMI+NaCl entered the apoptotic pathway; however, even assuming that the entire cell loss after 2 hr was due to the apoptosis only, the cumulative fraction of apoptotic cells was just 16%. In contrast, the same calculation for sucrose-protected cells yields over 50% of apoptotic cells. Thus, the primary necrosis was the predominant cell death pathway unless cells were protected with sucrose. For the data in Fig. 8, the primary necrosis was responsible for about 87% of the cell loss, versus 43% in the presence of sucrose.

Fig. 9 shows the time dynamics of cell subpopulations permeable to either YO-PRO-1, or both PI and YO-PRO-1, or not permeable to any of the dyes. Notably, YO-PRO-1 is both a sensitive indicator of membrane nanoelectroporation [25], [39], [41] and a marker of selective membrane permeabilization early in the course of apoptosis [42]. In contrast, the uptake of PI through nanopores is minimal; with the employed method of PI detection, its uptake manifests the irreversible cell destruction, by either primary or secondary necrosis.

Nanopores created by nsPEF still remained permeable to YO-PRO-1 at 20 min after the exposure, as seen by YO-PRO-1 uptake by most cells. In the RPMI+NaCl medium, these cells swelled and got destroyed (became PI-permeable) already within an hour. In the RPMI+sucrose group, many cells remained permeable just to YO-PRO-1 for several hours after the nsPEF exposure. The fraction of PI-positive cells was much smaller and stable at 1–4 hr after the exposure, followed by a delayed increase by 6 hr.

## Discussion

While it is widely accepted that nsPEF-exposed cells die by apoptosis, our results demonstrate for the first time that the primary necrosis was the predominant cell death mode. We also established that necrosis results from plasma membrane permeabilization, followed by water uptake, cell swelling, and eventual membrane rupture. This necrotic pathway is similar to what is seen with “classic” electroporation pulses or when applying various other necrotic factors.

This result may appear contradictory to the prevalence of nsPEF-induced apoptosis as reported by multiple other studies [1], [3], [4], [10], [14], [43]. However, these reports were based primarily on the flow cytometry counts of cells that display PS externalization; as discussed above, the relevance of this parameter to apoptosis in nsPEF-treated cells is questionable.

Although a number of studies reported evidence for necrotic cell death after nsPEF [4], [6], [9], [17], [28], this pathway has received little attention. Most of research focused on cellular mechanisms of nsPEF-induced apoptosis, whereas necrosis was viewed as a less common and less important event. In this study, for the first time we report that under standard cell culture conditions necrosis can be a prevalent mode of cell death. This finding holds true for rather diverse nsPEF exposure conditions, including different pulse durations, E-field values, and pulse delivery protocols.

With that said, the balance between apoptosis and necrosis can be profoundly dependent on the cell type and on the cell environment. For example, cells that do not have a large “stock” of spare membrane to swell will have less time for membrane repair after nsPEF, and are more likely to die from the membrane rupture. This fact may explain why smaller Jurkat cells were more vulnerable than larger U937 [12]. Cells within tissues *in vivo* may have limited room for swelling. Instead of the presence of sucrose, swelling can potentially be limited by the space constraints, thereby shifting the *in vivo* cell death towards apoptosis.

The profound increase of apoptosis in nsPEF-treated cells in the presence of sucrose raises a question if sucrose just “unmasked” the latent apoptosis or also facilitated the apoptotic cell death. For example, in Fig. 9 (right panel, RPMI+sucrose), the pool of YO-PRO-1 positive cells remained large for several hours after the exposure. This pool concurrently shrunk due to both resealing of nanopores and cell death, and expanded due to the development of apoptosis. One may speculate that the presence of sucrose could somehow inhibit the cell membrane repair, thereby leaving it permeable to YO-PRO-1 for longer time. Such long-lasting membrane disruption due to the impaired repair would be a plausible explanation for the onset of apoptosis in sucrose-protected cells; however, this mechanism does not appear to be supported by the data. Indeed, a large increase in the fraction of cells that did not uptake any of the dyes (between 0.3 and 2 hr) argued for the successful pore resealing in the RPMI+sucrose group. Therefore the development of apoptosis was not a side effect of the sucrose; instead, it was an effect of nsPEF exposure itself, which was masked by the faster necrotic process under the normal cell culture conditions.

The fact that nsPEF triggers both necrotic and apoptotic death mechanisms makes it an attractive modality for cancer ablation. First, the concurrent induction of both cell death modes reduces the chance for malignant cells to escape the death sentence, despite the sophisticated death evasion mechanisms developed by various cancers. Second, varying the nsPEF exposure parameters and combining nsPEF with sucrose injection or a similar treatment is an approach to deliberately induce either the apoptotic or necrotic death, or a combination thereof. For an *in vivo* cancer treatment, a carefully tuned combination of necrotic and apoptotic cell death may be an optimal solution for tumor elimination without excessive pain and inflammation while stimulating the immunogenicity of tumor cells [2], [44].
